# Associated infectious diseases in psychiatric patients who are hospitalized for long periods in a hospital in Braila

**DOI:** 10.1186/1471-2334-14-S7-O17

**Published:** 2014-10-15

**Authors:** Delia Mihaela Rasnoveanu

**Affiliations:** 1 Faculty of Medicine and Pharmacy, University Dunărea de Jos, Galați, Romania

## Background

Adult patients with severe psychical diseases are hospitalized in a separate building (ward) for very long periods of time (years). This ward has 140 beds and belongs to the Psychiatric Hospital “Sf.Pantelimon” in Brăila. Sometimes they live here for their entire life. In this situation they can develop several diseases, infectious and non-infectious, which need a special medical survey to prevent epidemics or complicated diseases.

## Methods

I studied 140 patients hospitalized in this ward in a period of 5 months (March to July 2014). I studied medical documents and I directly observed these patients. The eldest patient had spent 45 years in this institution and the most recent, 5 months. The oldest was 82 years old and the youngest was 21 years old. I studied the methods used for cleaning and disinfection (for people and environment), the substances used and working protocols for the prevention and control of nosocomial infections.

## Results

I discovered with a frequency lower than expected, different infectious diseases: respiratory (tuberculosis, acute respiratory illness of the upper and inferior tract), digestive (frequent gastro-enterocolitis, intestinal parasitosis), skin infectious (posttraumatic cellulitis, infections because of lying down for long time, herpes zoster), rare urinary tract infections. The problem is because these kind of patients often exaggerate (over or under) the symptoms, or may become immune to some germs in time. Sanitary alcohol and alcohol-based substances are very strictly used only by medical staff, because of the risk for alcohol addicted patients. Hygiene rules are different from other acute wards because of these patients’ characteristics.

## Conclusion

Associated infectious diseases in psychiatric patients who are hospitalized for very long periods are quite rare, even if the risk is permanent. Medical staff adapted hygienic and epidemiological rules to the patients and to special conditions. I think it is necessary to adapt the sanitary rules – by collaboration of epidemiologist, infectious disease and psychiatric specialists – to make easier the identification of diseases that could be contagious in such a hospital.

